# The role of haematological traits in risk of ischaemic stroke and its subtypes

**DOI:** 10.1093/brain/awz362

**Published:** 2019-11-22

**Authors:** Eric L Harshfield, Matthew C Sims, Matthew Traylor, Willem H Ouwehand, Hugh S Markus

**Affiliations:** 1 Stroke Research Group, Department of Clinical Neurosciences, University of Cambridge, Cambridge, UK; 2 Department of Haematology, University of Cambridge, Cambridge, UK; 3 Oxford Haemophilia and Thrombosis Centre, Oxford University Hospitals NHS Foundation Trust, NIHR Oxford Biomedical Research Centre, Oxford, UK; 4 Clinical Pharmacology, William Harvey Research Institute, Barts and The London School of Medicine and Dentistry, Queen Mary University of London, London, UK; 5 National Health Service (NHS) Blood and Transplant, Cambridge Biomedical Campus, Cambridge, UK; 6 British Heart Foundation Cambridge Centre of Research Excellence, University of Cambridge, Cambridge, UK; 7 Department of Human Genetics, Wellcome Trust Sanger Institute, Wellcome Trust Genome Campus, Hinxton, UK; 8 National Institute for Health Research Blood and Transplant Research Unit in Donor Health and Genomics, Department of Public Health and Primary Care, University of Cambridge, Cambridge, UK

**Keywords:** Mendelian randomization, haematology, clotting cascade, stroke, genetics

## Abstract

Thrombosis and platelet activation play a central role in stroke pathogenesis, and antiplatelet and anticoagulant therapies are central to stroke prevention. However, whether haematological traits contribute equally to all ischaemic stroke subtypes is uncertain. Furthermore, identification of associations with new traits may offer novel treatment opportunities. The aim of this research was to ascertain causal relationships between a wide range of haematological traits and ischaemic stroke and its subtypes. We obtained summary statistics from 27 published genome-wide association studies of haematological traits involving over 375 000 individuals, and genetic associations with stroke from the MEGASTROKE Consortium (*n* = 67 000 stroke cases). Using two-sample Mendelian randomization we analysed the association of genetically elevated levels of 36 blood cell traits (platelets, mature/immature red cells, and myeloid/lymphoid/compound white cells) and 49 haemostasis traits (including clotting cascade factors and markers of platelet function) with risk of developing ischaemic (AIS), cardioembolic (CES), large artery (LAS), and small vessel stroke (SVS). Several factors on the intrinsic clotting pathway were significantly associated (*P* < 3.85 × 10^−4^) with CES and LAS, but not with SVS (e.g. reduced factor VIII activity with AIS/CES/LAS; raised factor VIII antigen with AIS/CES; and increased factor XI activity with AIS/CES). On the common pathway, increased gamma (γ′) fibrinogen was significantly associated with AIS/CES. Furthermore, elevated plateletcrit was significantly associated with AIS/CES, eosinophil percentage of white cells with LAS, and thrombin-activatable fibrinolysis inhibitor activation peptide antigen with AIS. We also conducted a follow-up analysis in UK Biobank, which showed that amongst individuals with atrial fibrillation, those with genetically lower levels of factor XI are at reduced risk of AIS compared to those with normal levels of factor XI. These results implicate components of the intrinsic and common pathways of the clotting cascade, as well as several other haematological traits, in the pathogenesis of CES and possibly LAS, but not SVS. The lack of associations with SVS suggests thrombosis may be less important for this stroke subtype. Plateletcrit and factor XI are potentially tractable new targets for secondary prevention of ischaemic stroke, while factor VIII and γ′ fibrinogen require further population-based studies to ascertain their possible aetiological roles.

## Teaser

Whether haematological traits contribute equally to all ischaemic stroke subtypes is unclear. By analysing summary statistics from genome-wide association studies of haematological traits and MEGASTROKE, Harshfield *et al.* show that genetically altered levels of several coagulation factors are associated with ischaemic, cardioembolic, and large-artery stroke, but not small-vessel stroke.

## Introduction

Stroke is the second leading cause of death and disability-adjusted life years worldwide ([Bibr awz362-B20][Bibr awz362-B21]). Stroke represents a syndrome rather than a single disease, and is caused by a number of distinct pathologies. The majority of strokes are caused by cerebral ischaemia resulting in infarction, the most common subtypes of which are large-artery atherosclerotic stroke (LAS), cardioembolic stroke (CES), and lacunar stroke caused by cerebral small vessel disease (SVS) (Markus, 2016).

Blood cell traits, clotting factors, and platelet activation and aggregation pathways are involved in thrombosis and play a central role in the pathogenesis of stroke (Gregg and Goldschmidt-Clermont, 2003). Antithrombotic therapy with both antiplatelet agents and anticoagulants is widely used in the prevention of stroke (Gregg and Goldschmidt-Clermont, 2003), and genetic loci associated with risk of stroke are significantly enriched in targets for antithrombotics (Malik *et al.*, 2018). However, the mechanisms by which disorders associated with these haematological traits and haemostatic pathways result in ischaemic stroke is not fully understood. This information is critical for informing the development of new therapeutic targets for stroke prevention and treatment.

An important question regarding the optimization of secondary prevention treatment is whether thrombosis is an important disease mechanism in the major ischaemic stroke subtypes. Thrombosis and subsequent embolism play a key role in the pathogenesis of both CES and LAS, although trial data demonstrate that anticoagulants are more effective for CES and antiplatelet agents for LAS, suggesting the underlying thrombotic processes differ (Kapil *et al.*, 2017). Whether thrombosis plays an important role in SVS is unknown.

It has been suggested that activation of the coagulation system is a consequence of stroke itself (Brouns and De Deyn, 2009), and therefore determining whether altered coagulation is the cause or consequence of stroke is challenging. One way of overcoming this limitation is the use of Mendelian randomization; this technique is increasingly used to infer causality between exposure and disease through the use of genetic variants associated with the exposure as instrumental variables (Burgess *et al.*, 2018). The use of genetic variants, which are randomly allocated during meiosis, avoids reverse causality and minimizes confounding by environmental factors in a manner analogous to a randomized controlled trial (Burgess *et al.*, 2012).

Recent studies have provided large resources to examine the causal role of haematological traits in ischaemic stroke and its subtypes. The recent MEGASTROKE Consortium provided genome-wide association results from over 67 000 stroke cases and 450 000 controls (Malik *et al.*, 2018). Summary statistics from genome-wide association studies (GWAS) of numerous haematological traits, which in this paper refers to both blood cells (e.g. platelet count) and haemostasis parameters (e.g. factor VIII activity), are also available (Supplementary Table 1). We used two-sample Mendelian randomization, which combines published evidence from different data sources to obtain an estimate of the causal effect (Burgess *et al.*, 2015), to perform a comprehensive analysis of whether perturbations in haematological traits are implicated in stroke risk, and specifically whether certain stroke subtypes cluster with particular haematological traits.

## Materials and methods

### Data sources

#### Genetic associations with haematological traits

**Figure 1:**
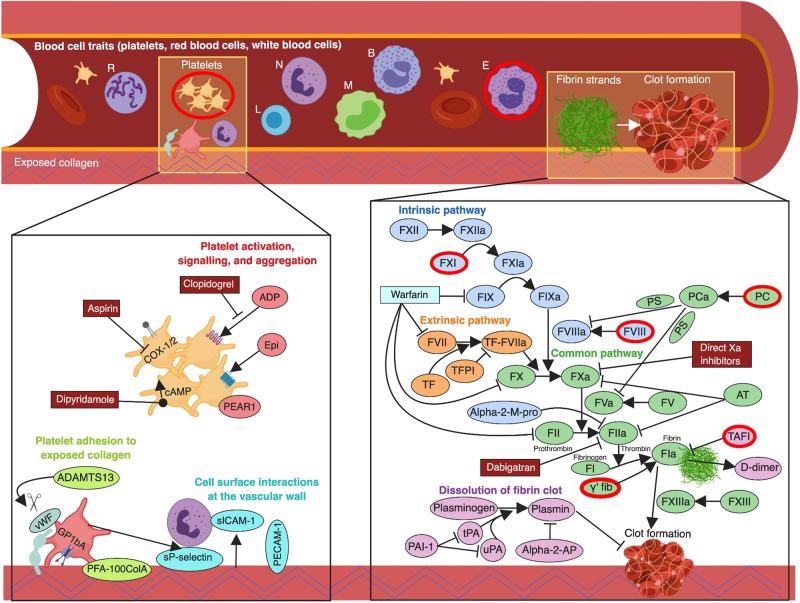
**Overview of haematological pathways and targets for stroke treatment and prevention.** arrow = agonize; B = basophils; E = eosinophils; L = lymphocytes; M = monocytes; N = neutrophils; perpendicular symbol = antagonize; R = reticulocytes; scissors = proteolytic cleavage. Traits significantly associated with one or more stroke subtypes are shown with a red border. Created with BioRender (https://biorender.com/).

We performed analyses using both blood cell traits (derived from a routine full blood count analysis), and haemostasis traits relating to the coagulation cascade, fibrinolysis, platelet function, and cell surface interactions at the vascular wall. An overview of the relationship between haematological traits and the various biological pathways involved is shown in Fig. 1.

We obtained genetic association estimates for blood cell traits from a recent publication on the largest GWAS of blood cell traits published to date, which involved 36 blood cell traits measured in 173 480 participants of European ancestry from the UK Biobank and INTERVAL studies (Astle *et al.*, 2016). The traits were classified under six categories: (i) platelets; (ii) mature red cells; (ii) immature red cells; (iv) myeloid white cells; (v) lymphoid white cells; and (vi) compound white cells.

To identify traits relevant to haemostasis we conducted a systematic review of the literature in PubMed (last updated 2 February 2019) searching for GWAS of all applicable traits. Details of the search strategy are provided in the Supplementary material (Appendix 1) and Supplementary Fig. 1. In circumstances where GWAS results for the same trait were available from more than one study, we used summary statistics from the study with the largest sample size. The studies for all traits included in the analysis had made available GWAS summary statistics in the public domain. The number of participants and genetic instruments available from each study are summarized in Supplementary Table 1, and details of the assay methods used by each study are provided in Supplementary Table 2. All methods with available summary statistics were included for traits that were assayed using multiple methods (e.g. factor VIII activity and factor VIII antigen). We used the Reactome database (Fabregat *et al.*, 2018) to classify the haemostasis traits according to the following expertly curated pathways: (i) extrinsic pathway (tissue factor activation); (ii) intrinsic pathway (contact activation); (iii) common pathway; (iv) dissolution of fibrin clot; (v) platelet adhesion to exposed collagen; (vi) platelet activation, signalling, and aggregation; and (vii) cell surface interactions at the vascular wall. For traits that were not easily captured in a single pathway we categorized them based on the pathway that we felt most closely reflected the biology of the trait.

#### Genetic associations with stroke

We obtained genetic associations with five stroke outcomes [all stroke (AS), all ischaemic strokes (AIS), CES, LAS, and SVS] for two ancestral groupings (European-specific and transancestral meta-analysis) from the MEGASTROKE Consortium, which included data from 67 162 cases and 454 450 control subjects (Malik *et al.*, 2018). Stroke cases were defined based on WHO criteria (i.e. sudden onset neurological changes of presumed vascular origin lasting at least 24 h) with stroke subtypes classified according to the Trial of Org 10172 in Acute Stroke Treatment (TOAST) criteria (Adams *et al.*, 1993). The available summary statistics from MEGASTROKE included 60 341 cases with any ischaemic stroke regardless of subtype, of which 9006 were CES, 6688 were LAS, and 11 710 were SVS.

### Statistical analyses

Mendelian randomization analysis was used to examine whether haematological traits have a causal effect on the development of ischaemic stroke and/or subtypes of stroke. The key assumptions of Mendelian randomization are that the genetic variant(s) used as instrumental variables (i) are associated with the risk factor; (ii) are not associated with any confounders of the risk factor–outcome association; and (iii) are independently distributed from the outcome conditional on the risk factor and any confounders (Burgess *et al.*, 2018). Genetic variants associated with haematological traits were identified as above and effect estimates were obtained for each instrument and harmonized with the outcome data, ensuring that the effect of each single nucleotide polymorphism (SNP) on the exposure and on the outcome corresponded to the same allele. We performed linkage disequilibrium clumping to ensure that the instruments used for each exposure were independent by selecting only the SNP with the lowest *P*-value amongst all SNPs with a linkage disequilibrium *r*^2^ ≥ 0.001. An estimate of the causal effect was assessed using the inverse-variance weighted (IVW) method. To provide more robust estimates and seek to overcome potential pleiotropy, we performed sensitivity analyses using additional Mendelian randomization models, namely Mendelian randomization-Egger regression, the weighted median estimator, and the simple and weighted mode-based estimators, which facilitated comparison of the causal estimates under different assumptions and using different statistical approaches (Burgess *et al.*, 2018). Analyses were performed separately within the European and transancestral populations from MEGASTROKE. We applied a multiple-testing correction for 130 disease pathway comparisons (13 pathways × two populations × five outcomes), which resulted in a significance threshold of *P* < 3.85 × 10^−4^.

To assess the potential for latent pleiotropy, we performed bidirectional Mendelian randomization (also known as reverse Mendelian randomization) for the significantly associated haematological traits (Zheng *et al.*, 2017). Although we were not hypothesizing that ischaemic stroke may actually cause changes in levels of haematological traits, these sensitivity analyses allowed us to assess whether there were any additional factors that may be driving the associations. We also performed a phenome-wide association study (PheWAS) using PhenoScanner (Kamat *et al.*, 2019) to explore whether any of the SNPs significantly associated with both haematological traits and risk of stroke were also associated with other relevant risk factors for stroke and related diseases. This could provide evidence of horizontal pleiotropy, an alternative causal pathway from the SNP to the outcome other than via the haematological trait under investigation, which would violate the assumptions of Mendelian randomization (Burgess *et al.*, 2018).

In view of associations we identified for both factors VIII and XI with CES, we used longitudinal data from 502 616 participants from the UK Biobank to determine whether a strongly significant genetic variant associated with these traits altered the risk of future stroke. We estimated hazard ratios with 95% confidence intervals (CIs) using Cox proportional-hazards regression models adjusted for age and sex, comparing the association with ischaemic stroke risk in 3962 individuals with atrial fibrillation, the most frequent cause of CES (Wolf *et al.*, 1991; Marini *et al.*, 2005; Gretarsdottir *et al.*, 2008; Lubitz *et al.*, 2017; Ntaios *et al.*, 2018), to the association in 498 654 control subjects without atrial fibrillation.

Mendelian randomization analyses were performed in R version 3.4.4 (R Core Team, 2018) using the ‘TwoSampleMR’ package version 0.4.21 (Hemani *et al.*, 2018), and Cox proportional-hazards regression estimates were obtained using Stata version 15.1 (StataCorp, 2018). Two-sided *P*-values and 95% CIs are presented.

### Data availability

The GWAS summary statistics used to perform the analyses described in this study were obtained from publicly available published data. All data generated or analysed during this study are included in this published article and its Supplementary material.

## Results

**Figure 2:**
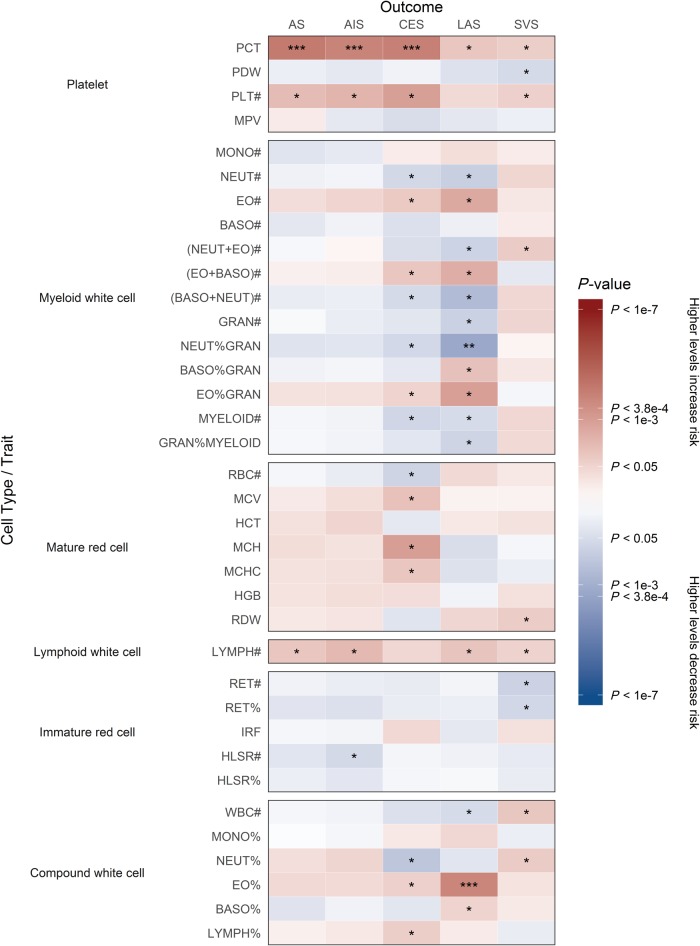
**Mendelian randomization results showing causal estimates for association of blood cell traits with stroke and its subtypes.** Mendelian randomization results are grouped by Reactome pathways according to blood cell traits: (i) platelets; (ii) mature red cells; (iii) immature red cells; (iv) myeloid white cells; (v) lymphoid white cells; and (vi) compound white cells. Refer to Supplementary Table 1 for a description of each trait. Colours show magnitude and direction of *P*-value of association for estimate of causal effect using inverse-variance weighted Mendelian randomization approach. Asterisks indicate the significance of the *P*-value for the most significant association (either European or transancestral population): *P < 0.05; ^**^P < 1 × 10^−3^; ^***^P < 3.85 × 10^−4^.

**Figure 3:**
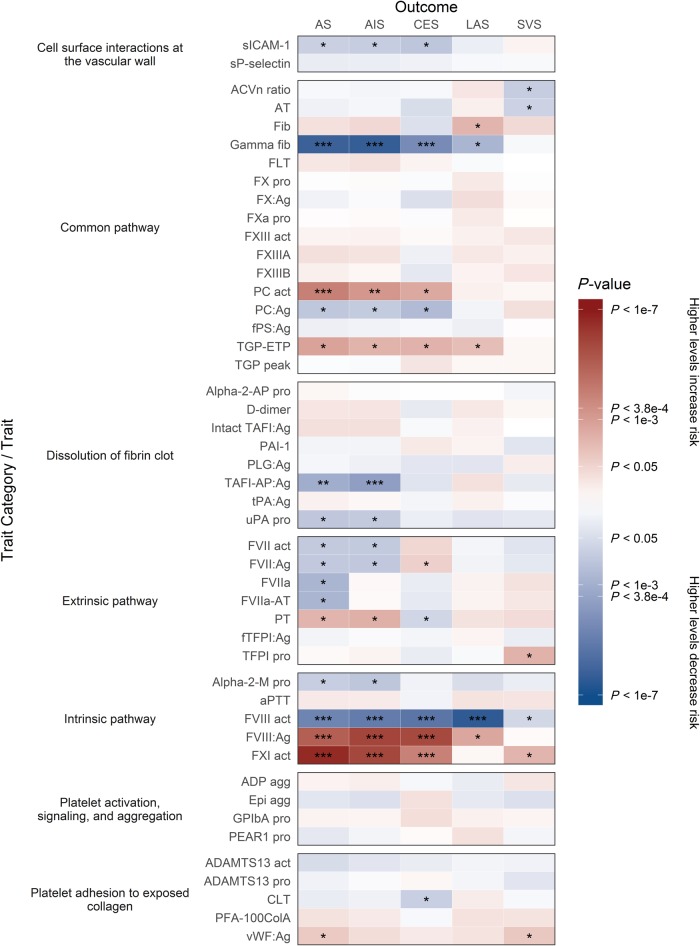
**Mendelian randomization results showing causal estimates for association of haemostasis traits with stroke and its subtypes.** Mendelian randomization results are grouped by Reactome pathways according to haemostasis traits: (i) extrinsic pathway of fibrin clot formation (tissue factor activation); (ii) intrinsic pathway of fibrin clot formation (contact activation); (iii) common pathway of fibrin clot formation; (iv) dissolution of fibrin clot; (v) platelet adhesion to exposed collagen; (vi) platelet activation, signalling, and aggregation; and (vii) cell surface interactions at the vascular wall. Refer to Supplementary Table 1 for a description of each trait. Colours show magnitude and direction of *P*-value of association for estimate of causal effect using inverse-variance weighted Mendelian randomization approach. Asterisks also indicate the significance of the *P*-value for the most significant association (either European or transancestral population): *P < 0.05; ^**^P < 1 × 10^−3^; ^***^P < 3.85 × 10^−4^.

**Figure 4:**
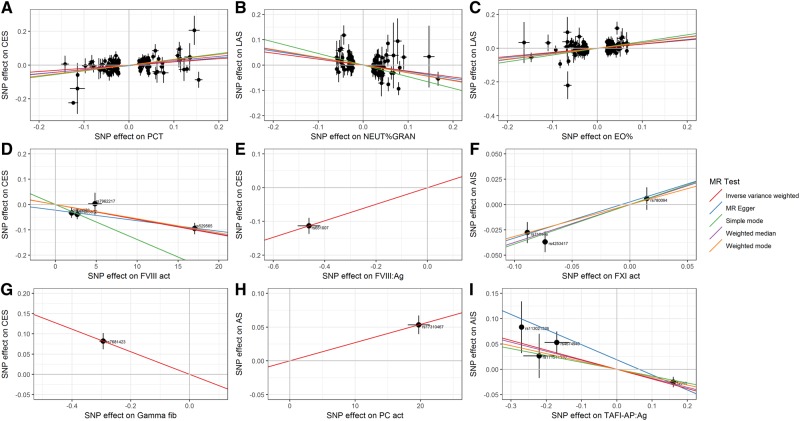
**Genetic associations with haematological traits and stroke subtypes with significant causal estimates.** The associations of each genetic variant associated with haematological traits with significant (*P* < 3.85 × 10^−4^) or marginally significant (*P* < 1 × 10^−3^) causal estimates are plotted against their association with selected stroke subtypes. Circles represent the associated change in levels of the trait and corresponding increased risk of stroke for each variant. The horizontal and vertical lines through each circle represent the corresponding 95% CIs for the genetic associations. Associations were oriented to the effect allele of each trait. Coloured lines show the slope (causal estimate) of the trait on stroke obtained using a variety of different Mendelian randomization (MR) approaches. Trait/outcome/population: (**A**) PCT/CES/European; (**B**) NEUT%GRAN/LAS/transancestral; (**C**) EO%/LAS/transancestral; (**D**) factor VIII activity/CES/European; (**E**) factor VIII antigen/CES/European; (**F**) factor XI activity/AIS/European; (**G**) γ′ fibrinogen/CES/transancestral; (**H**) protein C activity/CES/transancestral population; and (**I**) TAFI-AP:Ag/AIS/transancestral. See Supplementary Table 1 for a description of each trait.

From our systematic literature review of haematological traits we obtained published GWAS summary statistics for 36 blood cell traits and 49 haemostasis traits from 27 publications involving over 375 000 individuals (Supplementary Table 1) (Barbalic *et al.*, 2010; Johnson *et al.*, 2010; Smith *et al.*, 2010, 2011; Athanasiadis *et al.*, 2011; Lovely *et al.*, 2011; Paré *et al.*, 2011; Edelstein *et al.*, 2012; Huang *et al.*, 2012, 2014; Oudot-Mellakh *et al.*, 2012; de la Morena-Barrio *et al.*, 2013; Williams *et al.*, 2013; Ma *et al.*, 2014; Rocanin-Arjo *et al.*, 2014; de Vries *et al.*, 2015, 2016; Tang *et al.*, 2015; Astle *et al.*, 2016; van Loon *et al.*, 2016; Dennis *et al.*, 2017; Pankow *et al.*, 2017; Sennblad *et al.*, 2017; Kanai *et al.*, 2018; Olson *et al.*, 2018; Stanne *et al.*, 2018; Sun *et al.*, 2018). Figures 2 and 3 summarize the direction and magnitude of the association estimates for each haematological trait with each stroke subtype, with stronger associations indicated by darker colours (red in the positive direction and blue in the negative direction) and asterisks to indicate the level of statistical significance. Figure 4 shows scatterplots of the associations of each genetic variant plotted against their association with stroke subtypes for all traits that had significant (*P* < 3.85 × 10^−4^) or marginally significant (*P* < 1 × 10^−3^) associations: plateletcrit (PCT), neutrophil percentage of granulocytes (NEUT%GRAN), eosinophil percentage of white cells (EO%), factor VIII activity, factor VIII antigen, factor XI activity, gamma (γ′) fibrinogen, protein C (PC) activity, and thrombin-activatable fibrinolysis inhibitor activation peptide antigen (TAFI-AP:Ag). Figure 1 highlights significant associations from the Mendelian randomization analysis, provides a representation of the core components of each of the haematological pathways, and indicates existing drug targets for stroke prevention and treatment.

### Blood cell traits

Amongst the 36 blood cell traits, we observed the strongest associations for PCT (the volume fraction of blood occupied by platelets) and EO%. We found that genetically elevated levels of PCT were associated with significantly increased risk of AS, AIS, and CES in both European-only and transancestral populations (Figs 2, 4A and Supplementary Table 3), and increased EO% levels were significantly associated with LAS in both populations (Figs 2 and 4C). Decreased NEUT%GRAN levels were also marginally significantly associated with LAS in both populations (Figs 2 and 4B).

### Haemostasis traits

We examined three interconnected pathways of the clotting cascade. On the intrinsic (contact activation) pathway, factors VIII and XI were significantly associated with risk of stroke (Figs 3, 4D–F and Supplementary Table 4). Genetically elevated levels of factor VIII antigen and factor XI activity, and lower levels of factor VIII activity, were strongly associated with increased risk of AS, AIS, and CES, predominantly in both European-only and transancestral populations, and also with LAS in Europeans for factor VIII activity. Nearly all of the traits on the extrinsic (tissue factor activation) pathway had suggestive associations but were not statistically significant (Fig. 3).

On the common pathway, genetically lowered levels of γ′ fibrinogen (which contains the alternately spliced isoform of the fibrinogen gamma chain) were significantly associated with increased risk of AS, AIS, and CES in both populations (Figs 3 and 4G). Additionally, elevated protein C activity levels were significantly associated with increased risk of AS in both populations and marginally associated with AIS in a transancestral population, and there was suggestive evidence that it may also be associated with CES in both populations (Figs 3 and 4H). Elevated levels of total fibrinogen also exhibited suggestive evidence of association with LAS, though in the opposite direction to γ′ fibrinogen.

The remaining pathways that we examined were related to dissolution of fibrin clots (fibrinolysis); platelet adhesion to exposed collagen; platelet activation, signalling, and aggregation; and cell surface interactions at the vascular wall (Fig. 3). Genetically lowered levels of TAFI-AP:Ag, which is involved in the dissolution of fibrin clots, were significantly associated with increased risk of AIS in a transancestral population (Figs 3 and 4I).

**Figure 5:**
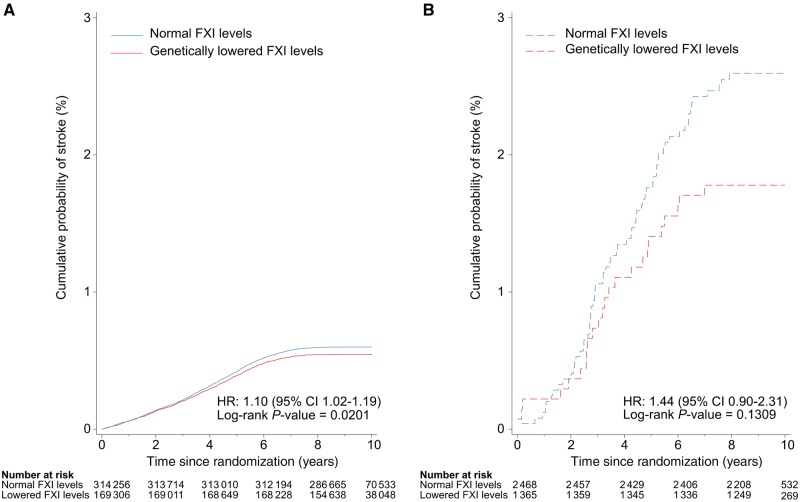
**Role of genetic variants associated with haematological traits in mediating association of atrial fibrillation with risk of ischaemic stroke.** Cumulative probability of ischaemic stroke in individuals with normal and genetically lowered levels of factor XI (FXI) activity (based on rs710446 alleles in *KNG1* locus) in UK Biobank participants (**A**) without atrial fibrillation and (**B**) with atrial fibrillation.

We also examined whether genetic variants significantly associated with factors VIII and XI were associated with risk of future stroke in atrial fibrillation cases and controls within the UK Biobank. rs710446 in the *KNG1* locus was selected as it was the SNP most strongly associated with factor XI activity and was also significantly associated with factor VIII activity. The hazard ratios for the association of rs710446 with ischaemic stroke were 1.44 (95% CI 0.90–2.31) in individuals with atrial fibrillation and 1.10 (95% CI 1.02–1.19) in controls without atrial fibrillation (Fig. 5). However, the absolute magnitude of the reduction in cumulative probability of stroke was much greater in individuals with atrial fibrillation, implying that treatments designed to reduce the risk of stroke by targeting factor VIII or factor XI levels are much more likely to be successful in patients with atrial fibrillation.

### Bidirectional Mendelian randomization and phenome-wide scan

In the reverse Mendelian randomization analyses, we found that AS and AIS were significantly associated with PCT, EO%, factor VIII activity, factor VIII antigen, factor XI activity, γ′ fibrinogen, and protein C activity. Many of these associations were found in both the European and transancestral populations and were confirmed using multiple Mendelian randomization methods (Supplementary Table 5). Our PheWAS found that the SNPs significantly associated with haematological traits and risk of stroke subtypes were also significantly associated with other relevant risk factors for stroke and related diseases, such as body mass index, systolic blood pressure, total cholesterol, LDL cholesterol, triglycerides, smoking status, coronary artery disease, and Alzheimer’s disease (Supplementary Tables 6 and 7).

## Discussion

This study provides a comprehensive description of the relationship between haematological traits and the risk of stroke and its subtypes. It highlights that a number of haematological traits are causal risk factors for stroke, but that these associations differ according to stroke subtype. The findings demonstrate that inter-individual genetic variation affecting several blood cell traits (PCT and EO%), components of the intrinsic (factors VIII and XI) and common (γ′ fibrinogen and PC activity) pathways of the clotting cascade, and a fibrinolysis inhibitor (TAFI-AP:Ag), significantly influence ischaemic stroke risk, particularly CES and (to a lesser extent) LAS, but not SVS.

Our findings have a number of clinical implications. First, they suggest that thrombosis, particularly due to abnormalities in the intrinsic and common pathways of the clotting cascade, plays an important role in the pathogenesis of both CES and LAS, but we found no associations with SVS. Embolism from cardiac pathology causes CES, while embolism from large-artery stenosis is believed to be the major pathogenic mechanism underlying LAS (Markus, 2016). Therefore, it is not surprising that genetic variants that increase thrombosis and thus increase the likelihood of formation of potential emboli are risk factors for both conditions. The role of thrombosis in SVS is much less certain. Case-control studies have suggested that platelet activation and coagulation may be less important for this subtype (Lavallée *et al.*, 2013), while increasing the intensity of antiplatelet therapy has not been shown to result in any further reduction of recurrent stroke in patients with SVS (SPS3 Investigators *et al.*, 2012). Furthermore, a previous candidate gene study showed the *ABO* locus, found to be associated with increased coagulation, was associated with CES and LAS but not SVS (Williams *et al.*, 2013). Taken together, these data raise the possibility that SVS is not driven by perturbations of platelets or the clotting cascade, implying that therapies designed to acutely dissolve thrombus (thrombolysis) or prevent thrombosis in the longer term (antithrombotic therapy) may not be effective in SVS. A further consideration is that small vessel pathology can cause both ischaemic SVS and deep intracerebral haemorrhage (Markus, 2016), meaning that optimizing the type and intensity of antithrombotic therapy is particularly important in this patient group. These hypotheses now need examining in randomized trials in well-phenotyped cohorts of patients with SVS.

Second, our analysis has identified new potential targets for pharmacological therapy and risk reduction of acute ischaemic stroke, particularly CES. Existing antiplatelet medication used to treat and prevent ischaemic stroke—including aspirin, clopidogrel, and dipyridamole—affect different signalling pathways involved in platelet activation and aggregation, thereby leading to reduced platelet function (Fig. 1). Our results suggest that reduction of PCT should also be considered. PCT is normally tightly controlled by the hormone thrombopoietin (TPO). TPO mimetics are drugs used for the treatment of immune thrombocytopenia, characterized by accelerated platelet clearance from the circulation and low platelet count and PCT (Kuter, 2009; Tang *et al.*, 2017). Therefore, a possible avenue for pharmacological research in acute ischaemic stroke would be the converse, i.e. antagonism of TPO or its haematopoietic stem cell receptor, MPL, in order to reduce PCT levels.

The intrinsic pathway of the clotting cascade is stimulated *in vivo* by methods including contact with negatively-charged polyphosphates released from activated platelets (Long *et al.*, 2016). It culminates in the coupling together of factor IX with its cofactor factor VIII to activate factor X. The only currently available anticoagulant used in stroke that acts on the intrinsic pathway is warfarin, which diminishes hepatic synthesis of factor IX as well as prothrombin (factor II) and factor X in the common pathway and factor VII in the extrinsic pathway. Disadvantages of warfarin include the inconvenience of regular blood tests for monitoring and annualized risk of major bleeding of ˜3% (Hylek *et al.*, 2014). This makes our finding that higher factor XI activity is significantly associated with an increased risk of AIS, and particularly CES, especially pertinent, as agents that reduce factor XI synthesis in the liver (Zhang *et al.*, 2010) and inhibit the action of factor XI’s activated form (factor XIa) (Lin *et al.*, 2006; Tucker *et al.*, 2009; Donkor *et al.*, 2017; Woodruff *et al.*, 2017) have already been developed. Phase I and II clinical trials of antisense oligonucleotides in several hundred individuals have shown that they reduce factor XI levels, have a major bleeding risk of only 0–1% (Liu *et al.*, 2011), and are superior in reducing radiologically detected asymptomatic deep vein thrombosis (Büller *et al.*, 2015). Our study extends the finding of a recent publication on the association of factor XI levels with AIS and CES in nearly four times as many stroke cases and 14 times as many control subjects (Gill *et al.*, 2018). Taken together with the early phase clinical trial data, there is now strong support for the need for randomized clinical trials of the use of factor XI inhibitors versus standard-of-care in ischaemic stroke, such as the international secondary stroke prevention trial using factor XIa inhibitor BMS-986177 (United States National Institutes of Health, 2019).

Our results indicate that factor VIII may also be a druggable target to consider for the prevention of both CES and LAS. To validate our findings, we demonstrated that the genetic variant most strongly associated with factor VIII activity and also associated with factor XI activity predicted risk of future stroke in patients with atrial fibrillation in the UK Biobank. Notably, the effect estimate was stronger in individuals with atrial fibrillation compared to those without atrial fibrillation, albeit the association in atrial fibrillation cases was not statistically significant, which is likely due to insufficient power. This suggests that drugs designed to inhibit factor XI activity (Bane and Gailani, 2014) or increase factor VIII activity might be effective at reducing the risk of ischaemic stroke in patients with atrial fibrillation, although studies with larger numbers of atrial fibrillation cases and randomized controlled trials would be needed to validate this finding. Additionally, the opposing direction of effect of factor VIII on stroke risk when measured by different assays means that further clarification of this finding in other population-based studies is required.

Through enzymatic cleavage of factor X to its activated form, factor Xa is the start of the common pathway that results in the generation of a fibrin clot. Existing anticoagulants used for secondary stroke prevention that affect this pathway include direct factor Xa and thrombin (factor IIa) inhibitors apixaban, rivaroxaban, and dabigatran (Fig. 1). However, our finding that increased levels of γ′ fibrinogen reduce the risk of AIS and CES would suggest that other distinct molecular pathways could potentially modify ischaemic stroke risk. γ′ fibrinogen results from the alternate splicing of one of the three genes (*FGG*), that is translated into fibrinogen and normally comprises 7% of circulating fibrinogen (Lovely *et al.*, 2011), and has distinct biochemical properties (Gersh *et al.*, 2009). After an acute ischaemic stroke, increased γ′ fibrinogen levels have been associated with higher scores for short-term disability (van den Herik *et al.*, 2011); however, the raised γ′ fibrinogen may reflect an acute phase response to stroke and it is difficult to ascribe causation (Farrell, 2012). Indeed, given that γ′ fibrinogen demonstrates defective platelet aggregation compared with fibrinogen (Farrell *et al.*, 1992), there is a biologically plausible mechanism by which γ′ fibrinogen is protective against ischaemic stroke that warrants further examination in prospective longitudinal studies.

One of the strengths of our analysis is that we show that several haematological traits are still strongly significant despite correcting for multiple statistical tests across all traits and outcomes. We only corrected for the number of disease–pathway comparisons analysed rather than the total number of unique traits, as this would have been too conservative due to correlations between many highly related traits. Some traits, such as factors X and XIII, were only measured in small studies (e.g. ˜2000 individuals or less), so they were penalized after the correction for multiple testing was applied and limited our power to detect associations with stroke. Likewise, there were approximately six times more cases available for all stroke than for subtypes of ischaemic stroke, so our power to detect statistically significant associations was also more limited for the ischaemic stroke subtypes (CES, LAS, and SVS). Additionally, many of the traits exhibited suggestive evidence (*P* < 0.05) that genetically altered levels of these traits could lead to increased risk of one or more stroke subtypes, though they did not reach the threshold for statistical significance after correction for multiple testing and therefore could be spurious findings. Further examination of more detailed associations between haematological traits and stroke subtypes than was accomplished in this analysis will only be feasible when larger samples sizes of well-phenotyped stroke cases become available.

Our study also has a few limitations. Our systematic review of GWAS of haematological studies was limited to studies listed in PubMed published in English, so there may be other studies that we missed. Additionally, two-sample Mendelian randomization should ideally be applied using non-overlapping datasets for obtaining genetic associations with the exposure and the outcome to reduce weak instrument bias (Burgess *et al.*, 2018). However, overlap between participants was unavoidable for some traits because the largest available published GWAS of those traits were measured in studies that were also included as part of the MEGASTROKE Consortium, such as the CHARGE Consortium, which comprised 10.7% of the total European stroke cases, and the BioBank Japan Project, which comprised 24.2% of the total non-European stroke cases (Malik *et al.*, 2018). Summary statistics from CHARGE and BioBank Japan were used to obtain genetic associations with the exposure for seven of the 85 traits. Moreover, there was significant heterogeneity amongst the different types of haemostasis traits that may limit their direct comparability. Several traits such as factor VIII antigen and protein C activity were only associated with a single independent SNP, so the causal estimates for these traits are less robust than for other traits that were associated with a large number of independent SNPs. Furthermore, the small number of participants included in some of the studies (e.g. 352 for antithrombin and 116 for PFA-100ColA) means that their genetic associations are less reliable than for the traits that were measured in tens or hundreds of thousands of individuals (e.g. 164 339 for PCT), which in turn limits the reliability of the causal inferences for these traits. Finally, the significant associations detected in the reverse Mendelian randomization analyses and PheWAS suggest that there is a possibility of horizontal pleiotropy if the outcomes were affected through other traits or pathways to those under investigation, so these findings should be interpreted with caution. Nevertheless, despite these limitations, we have applied a rigorous analysis approach to ensure our reported findings are as robust as possible.

In conclusion, we provide the first comprehensive analysis of the role of genetically elevated blood cell and haemostasis traits on risk of stroke. Of the 85 different traits that we analysed, PCT, EO%, factor VIII, factor XI, γ′ fibrinogen, protein C activity, and TAFI-AP:Ag have been identified as novel risk factors for ischaemic stroke, and we provide further support for the role of raised factor XI in its aetiology. Haematological traits also differentially associate with various stroke subtypes, suggesting the role of thrombosis may differ for different stroke subtypes. Methods for reducing PCT and factor XI levels should now be further examined in experimental and clinical research settings as potential new targets for secondary stroke prevention. We also demonstrate that this approach may identify potential drug targets for novel antithrombotic drugs.

## Supplementary Material

awz362_Supplementary_Data

## Abbreviations

AIS =: all ischaemic stroke
AS =: all stroke
CES =: cardioembolic stroke
EO% =: eosinophil percentage of white cells
GWAS =: genome-wide association study
LAS =: large artery stroke
PCT =: plateletcrit
SNP =: single nucleotide polymorphism
SVS =: small vessel stroke
TAFI-AP:Ag =: thrombin-activatable fibrinolysis inhibitor activation peptide antigen
